# Moderating effect of family structure on the relationship between early clinical exposure and emotional labor of nursing students: a cross-sectional study

**DOI:** 10.1186/s12912-024-02149-8

**Published:** 2024-09-02

**Authors:** Li Li, Ruiyang Xu, Shan Wang, Meng Zhao, Sijing Peng, Xinning Peng, Qingyuan Ye, Chen Wu, Kefang Wang

**Affiliations:** 1 School of Nursing and Rehabilitation, Shandong University, No. 44 Wenhua Xi Road, Lixia District, Jinan, Shandong 250012 China; 2 School of Nursing, Anhui University of Chinese Medicine, 350 Longzihu Road, Xinzhan District, Hefei, Anhui 230012 China

**Keywords:** Early clinical exposure, Emotional labor, Family structure, Moderating effect, Nursing students

## Abstract

**Background:**

Emotional labor is an essential component of nursing practice and is important for Generation Z nursing students born from the mid-1990s to early 2010s. They will become the backbone of the nursing workforce but present more emotional regulation problems. Studies on emotional labor are limited to clinical nurses and influencing factors at the individual level. The impacts of external systems on emotional labor of nursing students have not been explored. This study aimed to quantify the relationship between early clinical exposure and emotional labor and test the moderating effect of family structure on the relationship.

**Methods:**

The cross-sectional study recruited 467 nursing students using convenience sampling from seven colleges and universities in mainland China. An e-survey created on WJX.CN was used to collect data in January 2023. Emotional labor (surface acting and deep acting) was measured with the Emotional Labor scale. Early clinical exposure (exposure or not and times of exposure) and family structure (nuclear family, extended family, and single-parent family) were assessed with self-reported questions. Descriptive statistics and the linear mixed-effects modeling were used to do the analyses.

**Results:**

The mean scores of surface acting and deep acting were 26.66 ± 5.66 and 13.90 ± 2.40, respectively. A significant difference in scores of surface acting was not observed for exposure or not, whereas such a significant difference was found for times of exposure. Nursing students from extended families demonstrated significantly lower scores on surface acting while exposed to clinical practice compared with those from nuclear families. Family structure moderated the relationship between times of exposure and surface acting of nursing students when exposed to clinical practice for one time, but the significance disappeared when the times of exposure increased. No significant findings of early clinical exposure on deep acting were observed.

**Conclusions:**

Early clinical exposure influenced emotional labor, and students from extended families were more likely to get benefits from early clinical exposure. Studies are needed to help students from nuclear families get comparable benefits on emotional labor as those from extended families, and improve deep acting by early clinical exposure.

## Background

Emotional labor is an inevitable form of labor in nursing practice [1], and is the regulation of feelings and expressions to fulfill the interpersonal role expectations at work [2]. Emotional labor includes two emotional coping strategies: surface acting and deep acting. Surface acting is defined as the act of individuals trying to meet others’ expectations by suppressing negative and exaggerating positive expressions when interacting with people at work; deep acting is bringing feelings in line with observable expressions as required by display rules [3, 4]. Understanding the emotional labor among Generation Z (born from the mid-1990s to early 2010s) nursing students is particularly vital. Generation Z nursing students will become the backbone of the nursing workforce shortly [5]; in addition to requiring nurses to master proficient clinical skills, modern nursing work requires nurses to express appropriate emotion when communicating with patients, which is more emotionally challenging. Further, Generation Z was more likely (36%) than millennials (27%) and Generation X (20%) to report that their mental health and emotional well-being were as poor or only fair [6], and they present more emotional regulation problems under different conditions [7, 8].

Most studies on emotional labor were designed to quantify the impacts of emotional labor on clinical nurses’ health and work performances [9–12]; surface acting was found to be disadvantageous for nurses’ well-being [9, 13]and their professional performances [10], whereas deep acting was found to be constructive [10, 14]. Few studies were designed to explore influencing factors of emotional labor among nursing students. The human body is an open system, and individuals are exposed and influenced by various external systems, such as family, school, and workplace [15, 16]. Grandey et al. proposed in the model of emotional labor that external systems should be taken into consideration when investigating individuals’ emotional labor [3]. Therefore, we aimed to explore the impacts of representative external systems (i.e., school system and family system) on emotional labor of nursing students.

### Early clinical exposure and emotional labor

Undergraduates spent most of their time at school, and traditional education programs were designed to enrich their knowledge and skills, but limited information was designed to tailor to students’ emotional labor. Early clinical exposure may be taken as a candidate factor to understand the status of emotional labor. Early clinical exposure is a unique element in the school system for medical students, which fosters students to expose to the patients as early as the first year of medical college and includes teaching and learning activities such as observation, clinical bedside teaching and case-based learning lectures [17]. In China, early clinical exposure has been adopted into the training programs by some nursing schools in recent years.

Early clinical exposure brings some benefits and challenges to students in medical relevant education programs. Empirical evidence demonstrated that medical students in their first two or three years (i.e., the time when their learning is often from books or lectures in school) benefit from their encounters with patients [17], and the benefits include a better understanding of professional knowledge, and the enhancement of the clinical skills and professional attitudes [18]. A recent qualitative study also found that early clinical exposure may expose students to challenges that can evoke various strong emotions (i.e., bad, angry or scared), and nursing students would conduct surface acting when interacting with patients [19]. Furthermore, nursing students with higher scores of surface acting would have a stronger turnover intention in clinical practicum [20]. However, the effect of early clinical exposure on nursing students’ emotional labor remained unclear.

### Family structure and emotional labor

In addition to the school, family is a predominant system for cultivating individuals’ emotional regulation as individuals contact with their family of origin throughout their lives [21]. Family structure, an important variable of the family was found to be the significant contributor to emotional labor of individuals. Nuclear family, extended family and single-parent family are three common types of family structure. The nuclear family is often defined in literature as a family that consists only of parents and children [22]; the extended family is taken as an expansion of the nuclear family to a wider circle of relatives within the resident clan, and all the members should live close together, pool resources and undertake family responsibilities [23]; the single-parent family is comprised of a parent/caregiver and one or more dependent children without the presence and support of a spouse or adult partner who is sharing the responsibility of parenting [24]. When encountering emotional challenges, adolescents living with more family members would obtain more support, and that would empower them to regulate their feelings and expressions under different contexts; therefore, they would demonstrate more favorable emotional status [25–27]. For example, it was found that adolescents from extended families had less emotional problems and fewer risks of suffering from depression compared with those from nuclear families [25]; adolescents in nonnuclear homes were happier and less sad when interacting with older siblings or extended family members [26]. But within our knowledge, the relationship between family structure and emotional labor remained unclear among nursing students.

### Moderation effect of family structure

Existing studies limited studies to explore the association of factors in one system on individuals’ well-being while ignoring the interaction of factors of multiple systems. As proposed in the social-ecological model [16], there are multifaceted and interactive effects of systems and individuals. When students embark on their college/university education, school system is physically closer to students compared with family system, and the time of their interactions with school system is longer than that with their family system. Therefore, we aimed to explore the direct effect of school system (i.e., early clinical exposure) on emotional labor and the moderating effect of family system (i.e., family structure), and we proposed two hypotheses as follows.

#### Hypothesis 1

Early clinical exposure is significantly associated with emotional labor of nursing students.

#### Hypothesis 2

Family structure moderates the relationship between early clinical exposure and emotional labor of nursing students.

## Methods

### Design and sampling

We conducted a cross-sectional study with a convenience sampling strategy to collect data from students pursuing their bachelor’s degrees in the schools of nursing in mainland China. This study was launched in January 2023. Baccalaureate nursing education programs are typically four years in China. The first three years include courses on humanities character, social sciences, basic medicine and nursing, and students will start their internship in hospitals, community healthcare centers, and mental health centers in the fourth year. The inclusion criteria were full-time undergraduates enrolled in a four-year nursing education program; these students were in their first, second or third year of study and provided informed consent. Nursing students who have suspended their studies over six weeks for diseases or other reasons were excluded. According to Kendall’s sample size calculation method [28], the sample size is 5–10 times the number of independent variables, and this study used a total of 10 independent variables. Considering the loss of 20% samples, the sample size was 120 [*n* = 10 × 10 × (1 + 20%)].

### Measurements

Sample characteristics were assessed with a self-reported questionnaire. Age, sex (male/female), grade (freshman/sophomore/junior), single child (yes/no), and key decision maker on major selection (by myself/by my parents/by other relatives or friends/by the school) were assessed with close questions; video games play in daily life were assessed with open questions: “Do you play video games in daily life? What are they?”; nursing students who play interactive games that run on electronic media platforms, e.g., Honor of Kings, Counter-Strike: Global Offensive, League of Legends and Eggy Party in their daily lives were categorized as video gamers, and those left no response to these questions were categorized as non-video gamers.

Emotional labor was assessed with the Chinese version of the Emotional Labor scale [29]. This scale has 7 items to assess surface acting and 3 items to assess deep acting. Each item is graded on a 6-point Likert scale from 1 = strongly disagree to 6 = strongly agree. The higher sum score for each subscale indicates that individuals were more likely to act or display the corresponding emotional labor. The Chinese version of the Emotional Labor scale demonstrated satisfactory validity, and Cronbach’s α coefficients for surface acting and deep acting were 0.711 and 0.826, respectively [29].

Early clinical exposure was assessed with a self-reported questionnaire. In China, early clinical exposure was designed in some schools to bridge theoretical courses and clinical practice; it intersperses among the semesters or the vacations before the final-year internship, the schedule of which differs across schools; early clinical exposure once designed, students are mandatory to participate to get credits, and the predominant setting of exposure is the hospital. Guided by the interpretation of early clinical exposure proposed by Tayade and Latti [17] and the facts in China, we set up two open questions as follows to measure the early clinical exposure of nursing students.

1. Did you have a specialty practice in the hospital? (*thereafter*, exposure or not)
2. Times of hospital exposure (*thereafter*, times of exposure)

Family structure was assessed with one self-reported question “What was your family structure?” and responses were graded as nuclear family, extended family, and single-parent family with corresponding descriptions to assist in answering.

### Data collection

Seven medical colleges and universities were contacted for participation. Once the agreement was obtained from the director of the Office of Student Affairs, an e-survey created on WJX.CN along with a short descriptive text would be disseminated by students’ counselors to WeChat class groups. Nursing students could identify the link of the e-survey to respond to the questionnaire and were asked to provide informed consent at the first screen of the e-survey before proceeding. It takes approximately 10 min to complete the e-survey. A total of 559 responses were recorded for this study. After removing respondents who refused to participate (*n* = 89), 470 valid questionnaires were obtained.

### Data analysis

No outlier or missing value was detected in the data; we deleted the category of the single-parent family from the data because there were only 3 cases. Descriptive statistics were run for all variables. To assess the effect of early clinical exposure on students’ emotional labor, linear mixed-effects models were run, and each was used to regress one variable representative of early clinical exposure, family structure, and all sample characteristics (fixed effects) except school and grade (random effects) on surface acting or deep acting. In consideration of the cross-over interaction, an interaction term created by early clinical exposure × family structure was added to the model to estimate the significance of the moderation effect no matter whether the significant finding of the variable representative of early clinical exposure was observed in the reduced model. IBM SPSS Statistics Desktop 24.0 was used for all analyses. The effect size of each variable was estimated and reported with a 95% confidence interval (CI), and a *p*-value of lower than 0.05 was taken as statistically significant.

## Results

### Sample characteristics

In Tables 1 and 467 Generation Z nursing students were analyzed in this study. More than 50% of the students aged between 19 and 20 years old, and selected the major of nursing primarily by themselves. More than 80% of the students were female and lived in nuclear families, and more than two-thirds of them were not the single child of their parents. Almost 50% of the students were freshmen, and the majority of students (64.2%) enrolled in this study were not video gamers. There were 51% (238/467) of the students reported the experience of early clinical exposure, and of them, 49 and 49 exposed to hospitals 1 time and 2 times, respectively, and 140 reported the experience of exposing to hospitals 3 times or more. The average score of surface acting was 26.66 ± 5.66, while that of deep acting was 13.90 ± 2.40.

**Table 1:** Descriptions of sample characteristics (*n* = 467)

Characteristics	Categories	*N* (%) or Mean ± SD
Age	17-18years old	110 (23.6)
	19-20years old	237(50.7)
	21-23years old	120 (25.7)
Sex	Male	77 (16.5)
	Female	390(83.5)
Grade	Freshman	226 (48.4)
	Sophomore	83(17.8)
	Junior	158 (33.8)
Single child	No	324 (69.4)
	Yes	143(30.6)
Key decision maker on major selection	By myself	265 (56.7)
	By my parents	71(15.2)
	By other relatives or friends	69 (14.8)
	By the school	62 (13.3)
Video gamer	No	300 (64.2)
	Yes	167 (35.8)
Family structure	Extended family	57(12.2)
	Nuclear family	410 (87.8)
Early clinical exposure		
Exposure or not	No	229(49.0)
	Yes	238 (51.0)
Times of exposure	0	229 (49.0)
	1 time	49 (10.5)
	2 times	49 (10.5)
	3 times or more	140 (30.0)
Emotional labor	Surface acting	26.66 ± 5.66
	Deep acting	13.90 ± 2.40

Note: SD, standard deviation

### Effects of early clinical exposure on surface acting and moderating effects of family structure

**Fig. 1:**
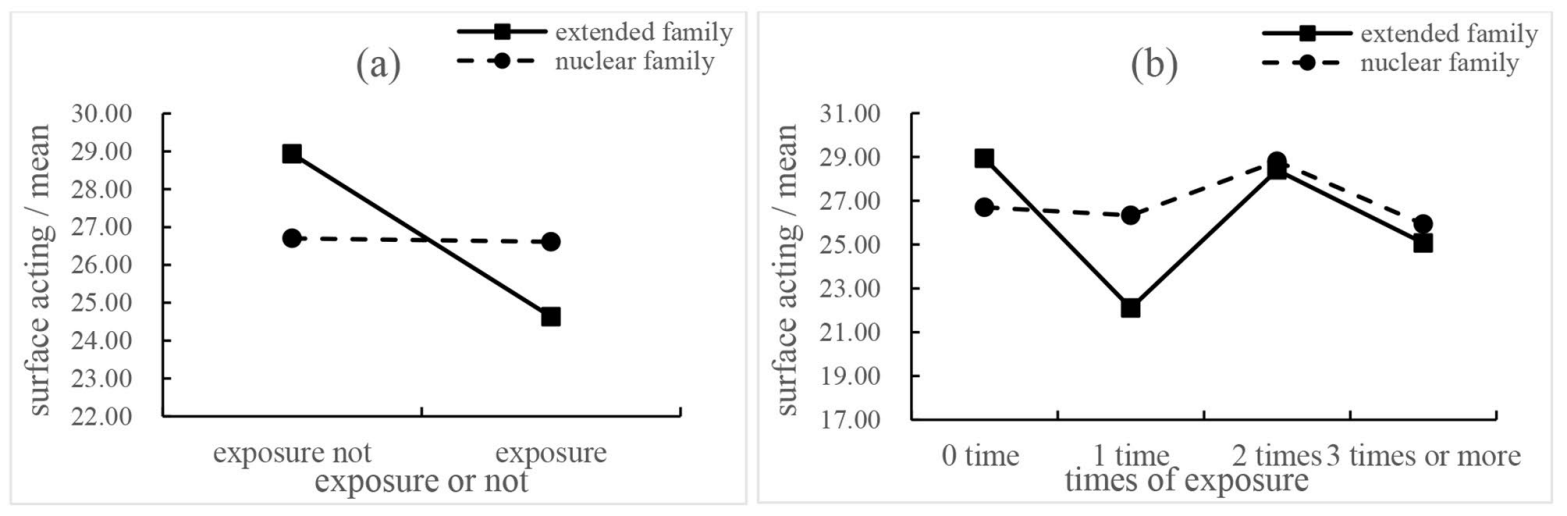
Moderating effects of family structure between early clinical exposure and surface acting of nursing students

As demonstrated in Tables 2 and 3, exposure or not had no significant effect on surface acting (*β* = -0.497, 95%CI [-1.931, 0.938], *p* = 0.494); times of exposure demonstrated a significant effect on surface acting (*p* = 0.045). When the interaction term of family structure × exposure or not was added to the model, we found students living in extended families would benefit more from early clinical exposure (*β* = -4.101, 95%CI [-7.219, -0.982], *p* = 0.010) compared with those living in nuclear families, i.e., their scores of surface acting decreased significantly after exposing to early clinical practice, see Table 4, Fig. 1**(a)**; meanwhile, the interaction term of family structure × times of exposure was found to be significant after being added to the model (*p* = 0.036), see Table 5. As shown in Fig. 1**(b)** and Table 5, students living in extended families demonstrated significantly lower scores on surface acting when exposed to clinical practice for one time compared with those living in nuclear families (*β* = -6.436, 95%CI [-10.921, -1.951], *p* = 0.005), but the significance disappeared when the times of exposure increased.

**Table 2:** The impacts of exposure or not on emotional labor (*n* = 467)

Variables	Surface acting					Deep acting				
	β	SE	*P*	LLCI	ULCI	β	SE	*P*	LLCI	ULCI
Age (ref. 17-18years old)			0.371					0.545		
19–20 years old	0.343	0.713	0.631	-1.059	1.745	0.303	0.297	0.309	-0.281	0.887
21–23 years old	-0.657	0.946	0.488	-2.518	1.203	0.380	0.387	0.326	-0.379	1.140
Sex (ref. Male)			0.338					0.520		
Female	0.741	0.771	0.338	-0.775	2.256	0.211	0.328	0.520	-0.433	0.855
Single child (ref. No)			0.437					0.177		
Yes	0.458	0.589	0.437	-0.699	1.615	-0.330	0.244	0.177	-0.811	0.150
Key decision maker on major selection (ref. By the school)			0.558					0.202		
By myself	-0.048	0.813	0.953	-1.646	1.550	0.677	0.341	0.048	0.006	1.348
By my parents	0.989	0.995	0.320	-0.965	2.944	0.278	0.419	0.507	-0.545	1.101
By other relatives or friends	0.448	1.004	0.656	-1.525	2.421	0.531	0.423	0.210	-0.301	1.363
Video gamer (ref. No)			0.160					0.619		
Yes	0.834	0.592	0.160	-0.330	1.998	-0.126	0.252	0.619	-0.621	0.370
Family structure (ref. Nuclear family)			0.915					0.394		
Extended family	0.086	0.803	0.915	-1.492	1.663	0.292	0.342	0.394	-0.381	0.964
Early clinical exposure										
Exposure or not (ref. No)			0.494					0.562		
Yes	-0.497	0.724	0.494	-1.931	0.938	-0.158	0.273	0.562	-0.696	0.379

Note: SE, standard error, LLCI, lower-level confidence interval, ULCI, upper-level confidence interval

**Table 3:** The impacts of times of exposure on emotional labor (*n* = 467)

Variables	Surface acting					Deep acting				
	β	SE	*P*	LLCI	ULCI	β	SE	*P*	LLCI	ULCI
Age (ref. 17-18years old)			0.479					0.624		
19–20 years old	0.428	0.707	0.545	-0.962	1.819	0.276	0.297	0.353	-0.308	0.861
21–23 years old	-0.386	0.945	0.683	-2.244	1.472	0.325	0.398	0.414	-0.457	1.107
Sex (ref. Male)			0.339					0.562		
Female	0.735	0.768	0.339	-0.775	2.244	0.190	0.328	0.562	-0.454	0.834
Single child (ref. No)			0.340					0.201		
Yes	0.559	0.585	0.340	-0.591	1.709	-0.313	0.244	0.201	-0.793	0.168
Key decision maker on major selection (ref. By the school)			0.682					0.220		
By myself	-0.094	0.807	0.908	-1.679	1.492	0.666	0.341	0.051	-0.004	1.336
By my parents	0.833	0.990	0.401	-1.114	2.779	0.286	0.420	0.496	-0.539	1.111
By other relatives or friends	0.141	1.003	0.888	-1.830	2.112	0.472	0.426	0.268	-0.364	1.309
Video gamer (ref. No)			0.167					0.609		
Yes	0.814	0.589	0.167	-0.343	1.972	-0.129	0.252	0.609	-0.624	0.366
Family structure (ref. Nuclear family)			0.813					0.309		
Extended family	0.189	0.802	0.813	-1.386	1.764	0.350	0.343	0.309	-0.325	1.024
Early clinical exposure										
Times of exposure (ref. 0)			0.045					0.320		
1 time	-1.276	0.986	0.197	-3.219	0.667	-0.670	0.399	0.094	-1.454	0.114
2 times	1.544	1.004	0.126	-0.440	3.528	0.140	0.397	0.725	-0.641	0.921
3 times or more	-1.160	0.871	0.188	-2.903	0.584	-0.050	0.325	0.878	-0.688	0.588

Note: SE, standard error, LLCI, lower-level confidence interval, ULCI, upper-level confidence interval

**Table 4:** The moderating effects of family structure between exposure or not and emotional labor (*n* = 467)

Variables	Surface acting					Deep acting				
	β	SE	*P*	LLCI	ULCI	β	SE	*P*	LLCI	ULCI
Family structure (ref. Nuclear family)			0.818					0.395		
Extended family	2.235	1.152	0.053	-0.030	4.499	0.300	0.495	0.545	-0.673	1.272
Early clinical exposure										
Exposure or not (ref. No)			0.030					0.664		
Yes	0.014	0.746	0.985	-1.464	1.492	-0.157	0.285	0.583	-0.717	0.404
Interaction items										
Family structure × Exposure or not			0.010					0.982		
Extended family × Exposure	-4.101	1.587	0.010	-7.219	-0.982	-0.015	0.683	0.982	-1.357	1.326

Note: SE, standard error, LLCI, lower-level confidence interval, ULCI, upper-level confidence interval;

Covariates: age, sex, single child, key decision maker on major selection, video gamer

**Table 5:** The moderating effects of family structure between times of exposure and emotional labor (*n* = 467)

Variables	Surface acting					Deep acting				
	β	SE	*P*	LLCI	ULCI	β	SE	*P*	LLCI	ULCI
Family structure (ref. Nuclear family)			0.405					0.484		
Extended family	2.263	1.146	0.049	0.011	4.515	0.306	0.495	0.537	-0.667	1.279
Early clinical exposure										
Times of exposure (ref. 0)			0.016					0.512		
1 time	-0.156	1.066	0.883	-2.257	1.944	-0.653	0.439	0.137	-1.515	0.209
2 times	1.848	1.042	0.078	-0.210	3.906	0.166	0.417	0.690	-0.653	0.986
3 times or more	-0.868	0.899	0.338	-2.663	0.928	-0.083	0.338	0.806	-0.747	0.581
Interaction items										
Family structure × times of exposure (ref. 0)			0.036					0.971		
Extended family × 1 time	-6.436	2.282	0.005	-10.921	-1.951	-0.062	0.987	0.950	-2.002	1.878
Extended family × 2 times	-2.908	2.882	0.313	-8.571	2.755	-0.283	1.245	0.820	-2.731	2.164
Extended family × 3 times or more	-2.907	1.908	0.128	-6.657	0.843	0.285	0.826	0.730	-1.337	1.908

Note: SE, standard error, LLCI, lower-level confidence interval, ULCI, upper-level confidence interval;

Covariates: age, sex, single child, key decision maker on major selection, video gamer

### Effects of early clinical exposure on deep acting and moderating effects of family structure

**Fig. 2:**
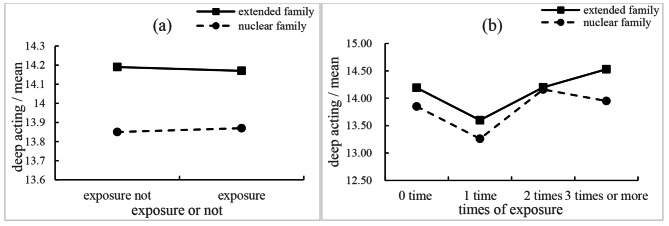
Moderating effects of family structure between early clinical exposure and deep acting of nursing students

As demonstrated in Tables 2 and 3, exposure or not had no significant effect on deep acting (*β* = -0.158, 95%CI [-0.696, 0.379], *p* = 0.562); times of exposure also had no significant effect on deep acting (*p* = 0.320). The effect of the interaction term of family structure × exposure or not was not significant when being added to the model (*β* = -0.015, 95%CI [-1.357, 1.326], *p* = 0.982), see Table 4, Fig. 2**(a)**; the effect of the interaction term of family structure × times of exposure was not significant when being added to the model (*p* = 0.971), see Table 5, Fig. 2**(b)**.

## Discussion

Emotional labor is often overlooked yet it is essential for nursing education, especially for Generation Z nursing students, as the nursing occupation is filled with emotional events, and emotional problems were frequently observed among this age cohort. This study was conducted to quantify the emotional labor of nursing students and investigate the impacts of variables from two closely related external systems, i.e., school and family on their emotional labor. We found some evidence to support the hypotheses that early clinical exposure was associated with emotional labor, and family structure moderated the relationship between early clinical exposure and emotional labor of nursing students.

Surface acting and deep acting are two compatible forms of emotional labor, which are conducted to respond to the service demands of patients and hospitals. Higher surface acting was a contributor to emotional exhaustion and depression [20, 30, 31], while higher deep acting would benefit individuals’ mental health [32]. Nursing students in this study demonstrated higher surface acting and lower deep acting in contrast with nurses working more than one year in tertiary hospitals [33], indicating that clinical environment may influence the development of individuals’ emotional labor.

In this study, we found exposure or not was not significantly associated with surface acting, yet times of exposure had a significant effect on surface acting. This further consolidated the findings of previous qualitative studies that early clinical exposure would evoke strong emotions and lead to emotional labor of students [19, 27]. Furthermore, family structure moderated the relationship, and students from extended families had lower surface acting than students from nuclear families once exposed to hospitals, that indicated students from extended families experienced more benefits from early clinical exposure. Specifically, students from extended families demonstrated reduced scores on surface acting when exposed to hospitals one time, two times, and 3 times or more, but that was not the case for students from nuclear families. Meanwhile, we found that the scores of surface acting of students from extended families were significantly lower than those among students from nuclear families during their first time of clinical exposure. Students may encounter unexpected emotional events (e.g., witness patients’ or their caregivers’ sorrow or hear stories of patients tortured by diseases) while exposed to the clinical setting; students from extended families would have more coping resources to buffer these clinical emotional challenges [34]. For example, extended family members might share some of their experiences with students to help them adapt to the emotional challenges [25, 27]. As such, students from extended families would be more likely to experience benefits. We did not capture the significant benefits along with the increase in the “dosage” of exposure, and this might be explained by that we did not investigate or take measures to balance the content of clinical exposure. Future studies may consider the content of early clinical exposure to elucidate the impacts of early clinical exposure on surface acting, and extra attention should be paid to students from nuclear families to understand how to help them get comparable benefits in reducing the scores of surface acting with those from extended families.

We failed to corroborate that early clinical exposure was significantly associated with deep acting in this study, nor did we find the moderation effect of family structure on such a relationship. Deep acting is a process where an individual psyches himself or herself to the desired emotion, which needs more emotional involvement [35]. In the literature, nursing students were found to prioritize learning procedural knowledge of different clinical tasks over learning how to interact with patients during early clinical exposure [36]. Some students reported that they would avoid deeply communicating with patients in poor conditions, such as cancer patients because they lacked of necessary communication skills and were fear of hurting patients [37]. These issues might explain the insignificant findings on the relationship between early clinical exposure and deep acting from this study. Future studies should explore complex interventions to deepen the involvement of nursing students in clinical exposure, such as developing strategies covering components of awareness raising, communication skills advancement, and encouraging deep interaction with patients during the exposure.

### Limitations

This study had several limitations. First, the inherited disadvantages including lack of sample representativeness and unable to make causal inferences of the cross-sectional study using convenience sampling strategy are nonnegligible. Future studies may want to launch cohort studies in representative samples to corroborate findings from this study. Second, family function is an important variable of family systems and may also influence the emotional labor of nursing students. We failed to address this variable in our study due to the diversity of its operationalizations across studies, and that its relationship with emotional labor has not been empirically identified. Meanwhile, family structure only included three common family types: nuclear family, extended family and single-parent family. Future studies may enroll students from other family structures, e.g., blended family and orphaned family, and assess the heterogeneity of their emotional labor. Third, we operationalized early clinical exposure as exposure or not and times of exposure, and one internship was considered as one exposure. However, exposure duration and exposure content might also be important parameters of early clinical exposure. Future researchers may want to measure high-resolution early clinical exposure and provide more sound evidence about the contributions of early clinical exposure to emotional labor of nursing students. Fourth, many other factors may influence individuals’ general emotional regulation including social interactions, physiological factors, and lifestyle choices, which may be potential influencing factors of emotional labor among nursing students, but the assessment of these variables is out of scope of this study. Future studies may want to collect data on these variables and use statistical methods such as the dominance analysis to present a comprehensive picture of factors associated with emotional labor.

## Conclusion

This study set out to verify the impacts of early clinical exposure and family structure on emotional labor of Generation Z nursing students. This study provided preliminary evidence supporting the significant contributions of early clinical exposure to surface acting, and the significant moderating role of family structure on this relationship. More efforts are needed to help students from nuclear families get benefits from early clinical exposure and to improve the deep acting of nursing students in general during nursing education.

## Data Availability

The datasets generated and/or analysed during the current study are not publicly available due to data privacy but are available from the corresponding author on reasonable request.

## Abbreviations

CI: Confidence Interval
